# Biodegradable Polymers in Veterinary Medicine—A Review

**DOI:** 10.3390/molecules29040883

**Published:** 2024-02-17

**Authors:** Magdalena Broda, Daniel J. Yelle, Katarzyna Serwańska-Leja

**Affiliations:** 1 Department of Wood Science and Thermal Techniques, Faculty of Forestry and Wood Technology, Poznan University of Life Sciences, Wojska Polskiego 28, 60-637 Poznan, Poland; 2 Forest Biopolymers Science and Engineering, Forest Products Laboratory, USDA Forest Service, One Gifford Pinchot Drive, Madison, WI 53726, USA; daniel.j.yelle@usda.gov; 3 Department of Animal Anatomy, Faculty of Veterinary Medicine and Animal Sciences, Poznan University of Life Sciences, Wojska Polskiego 71c, 60-625 Poznan, Poland; katarzyna.leja@up.poznan.pl; 4 Department of Sports Dietetics, Poznan University of Physical Education, 61-871 Poznan, Poland

**Keywords:** biopolymers, chitosan, polyhydroxyalkanoates, polycaprolactone, polylactic acid, natural polymers

## Abstract

During the past two decades, tremendous progress has been made in the development of biodegradable polymeric materials for various industrial applications, including human and veterinary medicine. They are promising alternatives to commonly used non-degradable polymers to combat the global plastic waste crisis. Among biodegradable polymers used, or potentially applicable to, veterinary medicine are natural polysaccharides, such as chitin, chitosan, and cellulose as well as various polyesters, including poly(ε-caprolactone), polylactic acid, poly(lactic-co-glycolic acid), and polyhydroxyalkanoates produced by bacteria. They can be used as implants, drug carriers, or biomaterials in tissue engineering and wound management. Their use in veterinary practice depends on their biocompatibility, inertness to living tissue, mechanical resistance, and sorption characteristics. They must be designed specifically to fit their purpose, whether it be: (1) facilitating new tissue growth and allowing for controlled interactions with living cells or cell-growth factors, (2) having mechanical properties that address functionality when applied as implants, or (3) having controlled degradability to deliver drugs to their targeted location when applied as drug-delivery vehicles. This paper aims to present recent developments in the research on biodegradable polymers in veterinary medicine and highlight the challenges and future perspectives in this area.

## 1. Introduction

Polymers are widely applied advanced materials that can be found in almost every product used in daily life. Nowadays, the importance of polymers has been much more highlighted because of their applications in different fields of science, modern technologies, and many branches of industry—from basic uses to biopolymers and therapeutic polymers.

The future of polymeric materials will rely heavily on their ability to biodegrade and return to the soil. To achieve this, the field of polymers has been shifting to organic-based materials for new syntheses and utilizing unique microorganisms to degrade existing persistent polymers, especially those that are not easily recycled. Polymers from biological materials may be sourced from polypeptides and polysaccharides with building blocks of amino acids and carbohydrates, respectively. Many biopolymers can be synthesized from these building blocks with the assistance of fermentation and microbial activity. Tremendous research efforts are also being made in the bioremediation of existing plastics through aerobic and anaerobic microorganism involvement, mineralizing them to CO_2_, H_2_O, and methane.

This paper aims to present recent developments in the application of biodegradable polymers in veterinary medicine and highlight the challenges and future perspectives in this area.

## 2. Polymers

The word “polymer”, or the sometimes-used “macromolecule”, is derived from the classical Greek “poly”, meaning “many”, and “meros”, meaning “parts”. The polymer molecule has a very high molecular weight (between 10,000–1,000,000 g mol^−1^). It consists of many smaller molecules named monomers, usually bound together by specific covalent bonds [1].

Polymers play a crucial role in human life because of their wide use in many industries. Generally, polymers have been around us in the natural world since the beginning; for example, cellulose from plant cell walls, starch from plant storage sugars, natural rubber from trees of the genus *Hevea*, and proteins in hide, wool, and silk. Manufactured polymeric materials have been studied since the middle of the 19th century, with studies on the building blocks of natural polymers, such as the elusive involvement of isoprene in rubber. The polymer industry has rapidly developed and is larger than the combined copper, steel, aluminum, and some other industries [1,2].

We usually do not comprehend that polymer-based products surround us every day, such as clothing made from synthetic fibers, fiberglass, polyethylene cups, nylon bearings, polymer-based paints, plastic bags, epoxy glues, polyurethane foam cushions, silicone heart valves, Teflon-coated cookware, and so on. The list is almost endless [3,4]. Moreover, the continuously developing technology allows for designing new generations of polymers with well-controlled architecture and microstructure, giving greater dimensions to their potential uses [5].

Polymers (biological, synthetic, and hybrid) also have a long history in medicine. Their uses range from traditional applications such as catheters, syringes, and blood-contacting extracorporeal devices to matrices for drug delivery, cell encapsulation, and tissue regeneration [6]. Table 1 presents examples of polymers most frequently used in medicine and their most common applications.

Such a wide variety of applications of polymers in broadly understood medicine makes it necessary to use mainly biodegradable polymers, the use of which will not contribute to increasing environmental pollution. Biodegradation, as a more advanced property of some polymers, finds application in an increasing number of fields, from suture materials via orthopedic stabilizing materials to vascular stents, because these devices may disappear after they have fulfilled their function. Responsive degradation of polymers upon defined triggers also allows controlled drug release applications. These concepts currently present the most active research fields, and products should soon appear in the medical device market [6,9].

## 3. Biodegradable Polymers

The basic elements of nature include carbon, hydrogen, oxygen, and nitrogen. These form the basis of living organisms and their biosynthesis, creating highly sophisticated biopolymers and hierarchal networks. Nature also has the ability to degrade these biopolymers and recycle their basic elements, returning them back to the soil, where they are once again available for new biosynthetic organisms. This cycle of life is continuous and constantly evolving with environmental changes, giving the earth life-sustaining oxygen and water.

Some fundamental concepts need to be discussed to understand the meaning of the term biodegradable. First, deterioration can be defined as the destructive alteration of a material that changes its chemical and physical properties, and there are distinct differences between biotic and abiotic deterioration agents. For example, those agents that are biotic derive from living organisms such as fungi, bacteria, invertebrates, and vertebrates, where nutrients are absorbed and digested by the biological organism. Abiotic agents are of non-living origin, such as ultraviolet radiation, thermal decomposition, chemical decomposition, freeze–thaw cycles, etc. [10].

Of the biotic agents involved in deterioration, fungi and bacteria are the most prevalent and use both enzymatic and non-enzymatic mechanisms to alter materials that serve as a food source. Currently, there is a great interest in harnessing the power of biotic agents to convert organic materials (e.g., waste in landfills or from industrial processes) into value-added products or to purposefully degrade highly recalcitrant polymeric materials so that they do not persist in the environment. This is where biodegradable polymers come into focus. The future of the polymer industry will depend on its ability to incorporate monomers sourced from waste material (including polymeric waste) and the ability of the newly synthesized polymers to eventually degrade from biotic agent activity, that is, to be biodegradable [11,12].

**Figure 1:**
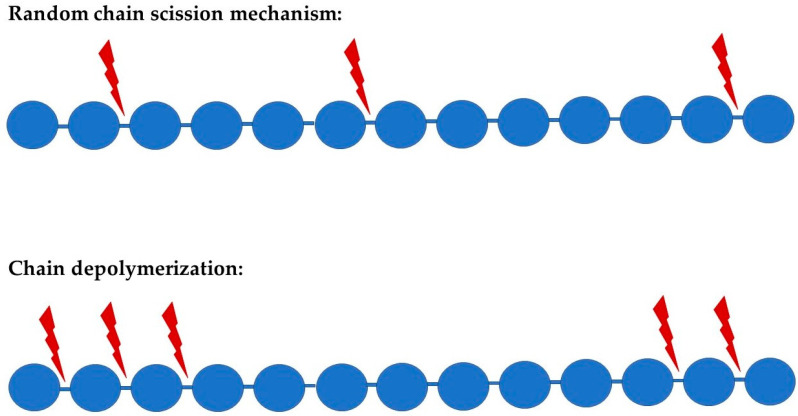
Schematic representation of the two principal suggested polymer degradation mechanism; red arrows represent scissions during the process.

A major proponent for polymers of any type to become depolymerized involves its equilibrium polymerization (or reverse polymerization). In the biodegradation of polymers, there are two principal degradation processes that might occur, which are random degradation and chain depolymerization (Figure 1). Random degradation, parallel to stepwise polymerization, occurs when there is rupture or scission at random points along a polymer chain. Since the scissions are random, large polymeric or oligomeric fragments are created. Chain depolymerization, parallel to chain polymerization, is a free radical process that occurs when monomeric units are released through depropagation, leading to a reaction similar to unzipping. These reactions may occur on their own or simultaneously with the biotic agent involved. Molecular weight can be used to differentiate these two processes in which random degradation yields a diverse mixture of higher molecular weight fragments and chain depolymerization yields principally monomers [12].

Random degradation may be initiated at a weak link in the polymer chain, such as in the case of ozonolysis of butyl rubber, where the initial attack is at the double bonds of the isoprene residues, or in the case of acid-catalyzed hydrolytic degradation of cellulose, where the number of chain ends increases linearly with time in a random fashion. In chain polymers, it is common for a certain “ceiling temperature” to trigger the reverse reaction, leading to a depropagation via unzipping. However, other mechanisms produced by chemical, photolytic, or enzymatic means may also cleave bonds in chain polymers, yielding an active end-group and depolymerization [13].

The susceptibility of various polymers to degradation can be imagined by categorizing them into how polar the bonds are and how the bonds are accessible to hydrolytic fluids. Williams [14] grouped the stability of polymers in the following way: (1) hydrophobic and not accessible; (2) hydrophilic and not accessible; (3) hydrophobic and accessible; and (4) hydrophilic and accessible.

Another approach to categorizing polymers as biomaterials is by solubility of the polymers in their respective physiological environment [15]. For drug binding and delivery vehicles, solubility must be high, but for transient implants or tissue scaffolds where polymers are in direct contact with living tissue, inertness is important, and thus solubility must be much lower. Therefore, the application will drive the type of polymer needed.

When water-insoluble (i.e., hydrophobic) polymers are considered, biodegradation becomes challenging. Making the polymer more accessible to water or the physiological fluids leads to an increase in swelling of the polymer matrices and may allow enzymes to infiltrate into the free volume so that degradation may commence. Biodegradation of hydrophobic polymers containing crystalline domains may require a mechanical disruption to proceed with further degradative processes [15].

In January 2018, the European Union released its vision for a more sustainable plastics industry to be achieved by 2030. The development of biodegradable polymers has been on the upswing for several years as they promise solutions to existing problems: they are used in medicine and tissue engineering. Among other things, they are applied as carriers that deliver drugs more specifically to the target organ and release the drug after a specific stimulus. Biodegradable polymers are also promising alternatives to mostly non-degradable commodity polymers to combat the global plastic waste problem. Over 250 million tons of commodity plastics are produced worldwide annually [16].

In Poland, about 50 million m^3^ of communal waste is collected annually. Wastes from synthetic materials account for 10–13% of this, but their percentage is still increasing, increasing the surface of waste dumps. This causes severe danger to the natural environment because plastics accumulate in the environment, can be fragmented into smaller pieces (so-called micro-plastics) and migrate via rivers to the oceans, where they form accumulation zones—so-called garbage patches. One solution to the problem may be increasing the production and utilization of biodegradable polymers. In contrast to the majority of industrial polymers, biodegradable ones are supposed to decompose to water, carbon dioxide, and biomass once they end up in the environment [16]. The major advantages of biodegradable plastics are that they can be composted with organic wastes and returned to enrich the soil, as was mentioned above. Moreover, their use will not only reduce injuries to wild animals caused by the dumping of conventional plastics but also lower the labor cost of removing plastic wastes from the environment because they are degraded naturally. Furthermore, their decomposition will help increase landfills’ longevity and stability by reducing the garbage volume. Additionally, they could be recycled into valuable monomers and oligomers by microbial and enzymatic treatment and serve as raw materials for fabricating other products [17].

Conventional plastics typically have additives such as plasticizers or fillers that may leach into the surrounding physiological environment, or in the case of medicine into the living tissue and may evoke a toxic or inflammatory response [18]. Biopolymers are made partly or entirely from synthetically or naturally derived polymers. They are mainly composed of polysaccharides, proteins, and fibers. Individually, these biopolymers do not demonstrate similar material properties or mechanical strengths comparable to traditional plastics, which limits their direct use. Thus, enormous research and development activities to improve biopolymers have been undertaken to equalize their mechanical and physical properties with those of non-biodegradable polymers and enhance their applications [19,20].

Generally, commonly used biodegradable materials may be divided into three groups: the mixtures of synthetic polymers and substances which are easily digestible by microorganisms (e.g., modified natural polymers, natural polymers such as starch and cellulose), synthetic materials containing chemical groups that are susceptible to hydrolytic microbial attack (for example, polycaprolactone) and the biopolyesters sourced from bacteria. The most popular and important biodegradable polymers are aliphatic polyesters (e.g., polylactide, poly(ε-caprolactone), polyethylene oxide, poly(3-hydroxybutyrate), polyglycolic acid), thermoplastic proteins, and polyhydroxyalkanoates, including polyhydroxybutyrate [4,17,21].

Both natural and synthetic polymers are remarkably involved in the comfort and facilitation of human life. They are responsible for life itself, for example, nutrition, medication, communication, irrigation, transportation, containers, recording history, clothing, buildings, highways, and many others. In fact, it is not easy to imagine modern human society without synthetic and natural polymers [22].

## 4. Application of Biodegradable Polymers in Veterinary Medicine

**Figure 2:**
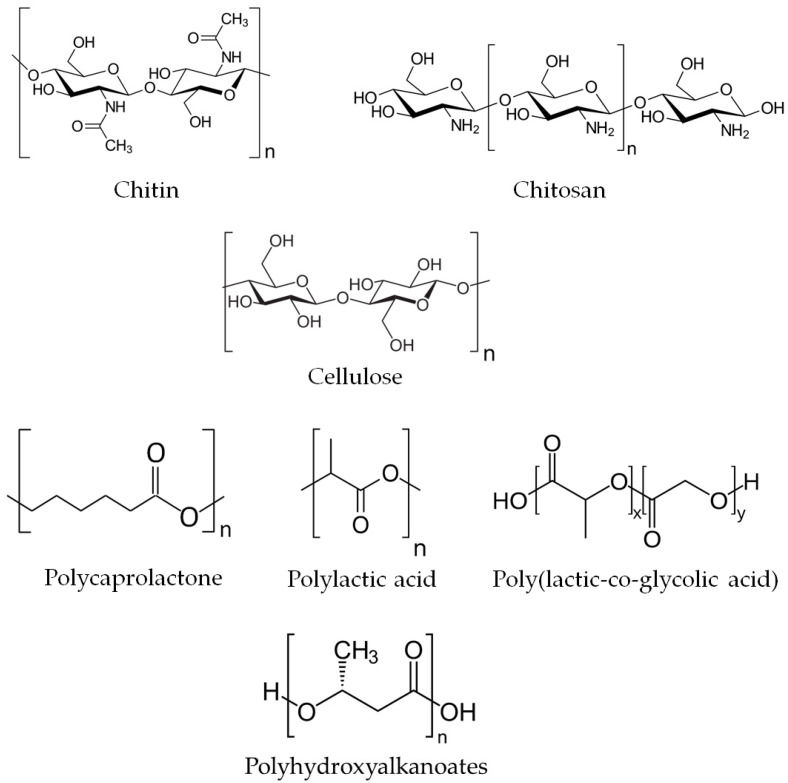
Chemical structure of the most common biodegradable polymers applied in veterinary medicine.

The most attractive biodegradable polymers used or potentially applicable in veterinary medicine are natural polysaccharides, such as chitin, chitosan, and cellulose, as well as various polyesters, including polycaprolactone, polylactic acid, poly(lactic-co-glycolic acid), and polyhydroxyalkanoates (Figure 2). They are available commercially in various forms (powder, beads, gel, fibers, films, etc.) and molecular weights, which facilitate specific applications and control of polymer degradation where necessary (e.g., in drug-delivery systems) [23].

The use of biopolymers in medicine depends on their biocompatibility, mechanical resistance, and sorptive characteristics. Today, they are most commonly used as implants in vascular and orthopedic surgery, for the production of materials such as catheters, products for gynecology and hemodialysis, tooth reconstruction, and so on. In pharmacy, they are used as a medicine matrix carrier to allow the controlled release of drugs within the body [24]. In recent years, there has been a growing interest in using biodegradable polymers in tissue engineering. In the design and development of tissue engineering products, the selection of biomaterials plays a key role. Biomaterial must interact with tissue to repair rather than act simply as a static replacement. Moreover, biomaterials used directly in tissue repair or replacement applications (e.g., artificial skin) must be more than biocompatible: they must elicit a desirable cellular response. Consequently, a major focus of biomaterials for tissue engineering applications revolves around harnessing control over cellular interactions with biomaterials, often including components to manipulate cellular response within the supporting biomaterial as a key design component. A tissue engineering product developer needs to have several biomaterial options available because each application calls for a unique environment for cell-cell interactions. Such applications include:

- Support for new tissue growth (wherein cell–cell communication and cell availability to nutrients, growth factors, and pharmaceutically active agents must be maximized);
- Prevention of cellular activity (when tissue growth, such as in surgically induced adhesions, is undesirable);
- Guided tissue response (enhancing a particular cellular response while inhibiting others);
- Enhancement of cell attachment and subsequent cellular activation (e.g., fibroblast attachment, proliferation, and production of an extracellular matrix of dermis repair);
- Inhibition of cellular attachment and/or activation (e.g., platelet attachment to a vascular graft); and
- Prevention of biological response (e.g., blocking antibodies against homograft or xenograft cells used in organ replacement therapies).

Biodegradable polymers are useful to those tissue engineering applications for which tissue repair or remodeling is the goal but not where long-term material stability is required. Biodegradable polymers must also possess:

- Manufacturing feasibility, including sufficient commercial feasibility;
- The capability to form the polymer into the final product design;
- Mechanical properties that adequately address short-term function and do not interfere with long-term function;
- Low or negligible toxicity of degradation products;
- Drug delivery compatibility in applications that call for the release or attachment of active compounds [25].

### 4.1. Chitin and Chitosan

Chitin is a natural homopolysaccharide composed of N-acetyl glucosamine units connected by β-(1,4) linkages (Figure 2) and is insoluble in water and other common solvents. It is the second-most abundant biopolymer after cellulose and a principal structural component of crustacean and insect exoskeletons and cell walls of some bacteria and fungi [26].

Chitosan is a partially deacetylated derivative of chitin obtained through alkaline deacetylation (Figure 2). Commercially, it is available as a powder, fibers, or microcrystals as well as in processed forms like beads, films, membranes, or microcapsules [27]. Due to its easy solubility in acidic aqueous media and versatile properties, chitosan is used in numerous applications in agriculture, food processing, papermaking, wood protection, waste and water treatment, fabric and textiles, cosmetics, and nutritional enhancement [28,29,30,31,32,33]. Chitosan is nontoxic and non-allergenic, bioactive, biocompatible and biodegradable. With its antimicrobial and hemostatic properties, it is a valuable chemical for several pharmaceutical and biomedical applications in both human and veterinary medicine [34,35,36,37,38,39].

Exceptional properties of chitosan result from its chemical structure. The presence of functional groups—including an amino group at C2, multiple primary hydroxyl groups at C6, and secondary hydroxyl groups at C3—enable interactions with other molecules through hydrogen bonds. In this way, chitosan molecules can be modified and not only form long polymers but also crosslink with other polymers, thus producing various co-polymers with new functionalities [40,41].

#### 4.1.1. Chitosan in Wound Management

Among potential biomaterials suitable for wound treatment of veterinary patients, chitosan is one of the most cost-efficient options. Chitosan hydrogels meet the essential requirements for wound dressings, such as low adhesion to the lesion surface, absorption of exudation, ability to exchange oxygen, and moisture promotion, which guarantees solubilization of antimicrobial agents and growth factors and facilitates fibroblast growth, thus stimulating healing [39,42].

**Figure 3:**
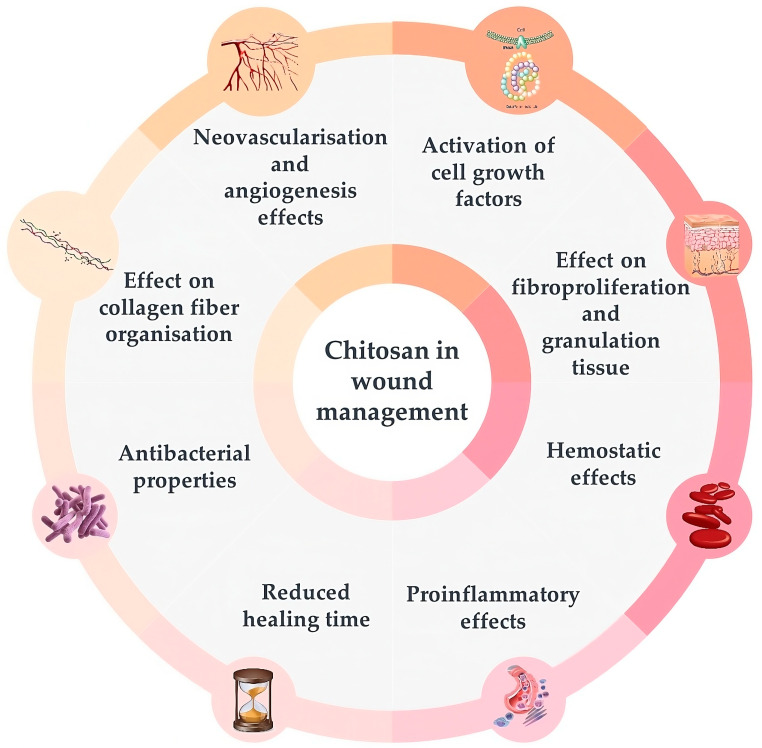
The activities of chitosan useful in wound management in animals – figure was prepared based on the information given in [39].

Chitosan films are hard but elastic and flexible, with excellent antimicrobial and wound healing properties with a low risk of toxicity and immune response, which makes them perfect as wound-dressing material [39,43,44,45]. Additionally, the chemical structure of chitosan with positively charged amino groups allows it to interact with several proteins, thus influencing various processes involved in wound healing. Several studies confirmed a variety of therapeutic effects of chitosan or its derivatives, such as N,N,N-trimethyl-chitosan (TMC), N,O-carboxymethyl-chitosan (CMC), and O-carboxymethyl-N,N,N-trimethyl-chitosan (CMTMC) individually or in combination with other agents on animal skin lesions (Figure 3) [39,42,46,47,48,49,50,51].

Chitosan attracts and activates cell growth factors when applied to wounds due to its electrostatic interactions with glycosaminoglycans [48,49]. It was shown to boost vascular endothelial growth factor (VEGF) and fibroblast growth factor (FGF-2) expression in animals by immunostaining and treatment with hydrocolloid sponges and chitosan-collagen sponges [50,52,53]. Moreover, chitosan hydrogels can serve as storage tanks for various proteins, including growth factors, which can be administered locally to enhance healing [53,54,55].

Chitosan also positively affects fibroproliferation and the increase of granulation tissue, which are necessary to reconstruct tissue architecture in the case of extensive wounds and severe distortions, such as complete excisional back wounds or first- and second-degree burns [53,54,56,57,58,59].

The hemostatic activity of chitosan has been reported in several trials, confirming its usefulness in managing injuries [60,61,62,63]. It attracts fibrinogen and plasma proteins involved in blood clotting. Positively charged amino groups of chitosan can bind with the negatively charged erythrocytes, causing hemagglutination [43,48].

The induction of immunological response by chitosan involves igniting mononuclear and polymorphonuclear cell migration to the wound bed [64,65]. The pro-inflammatory effects of chitosan were confirmed in several clinical trials with rats [54,66,67].

Chitosan in the form of gel, film, or nanofibers was also effective in reducing wound healing time, as confirmed in trials with rats when applied to excisional wounds on the back or second-degree burn injuries [56,68,69,70,71,72].

The presence of amino groups makes chitosan a cationic polymer that can strongly bind electrostatically to anionic proteins in the cytoplasmic membrane of bacterial cells, resulting in membrane rupture and death of the cell. Moreover, low molecular chitosan (<5 kDa) can penetrate bacterial cell walls and inhibit both DNA transcription and mRNA synthesis, precluding bacterial growth and activity. It also has chelating properties, thus inhibiting bacterial toxins [45]. Antimicrobial properties of chitosan have been reported in several clinical trials, including those with rats and cattle with wounds contaminated with *Staphylococcus aureus*, where a reduction in bacterial colonization was observed after applying 0.5 and 1% chitosan gel and film, as well as gels incorporated with chlorhexidine digluconate loaded chitosan nanoparticles [67,73,74].

Chitosan gels, films, and fluorinated methacrylamide chitosan hydrogel facilitate tissue repair and healing by improving the organization of collagen fibers in wounds, as shown in the example of excisional back wounds both clean and contaminated with *S. aureus* in rats [54,69,75,76].

Chitosan’s neovascularization and angiogenesis effects are additional benefits of using it on extensive wounds. Due to the activation of interleukine-1 controlling the proliferation of fibroblasts and collagen synthesis, chitosan accelerates the angiogenesis process and migration of neutrophils. It also increases vasculogenesis, as shown in trials with rats and horses [39,50,52,56,67,77].

The properties of chitosan that are useful for lesion treatment also include its anti-edematous effects [69], ability to improve keratinocyte proliferation and epithelial growth [56,68], as well as analgesic, antipruritic, antifungal, anti-biofilm, and antierythematous properties [39,48,69].

Importantly, none of the chitosan forms used in research (powder, gels, films, sponges) exhibited any adverse effects on animal skin and health in general; therefore, they are considered safe biomaterials for in vivo applications in veterinary medicine [48,56,57,58,69,73].

#### 4.1.2. Chitosan for Tissue Engineering

Scaffolds are fundamental in tissue engineering, being an excellent, continuously developed alternative to transplanting. They do not serve as tissue replacements in living organisms, but enable the adherence of specific cells and provide a favorable environment that facilitates proper cell differentiation and proliferation, ensuring the usefulness and adequate functioning of regenerated tissue [41,78,79,80].

The requirements for biomaterials used as scaffolds include biocompatibility, biodegradability, appropriate mechanical strength and the possibility to be easily shaped into different shapes and preserve the anatomical tissue volume and shape, lack of toxicity, allergic reactions or other side effects, controlled deliverability of bioactive molecules, proper porosity to enable adhesion of cells, and half-life long enough to ensure the growth of a specific tissue, and chitosan either alone or in combination with other agents meets them all [79,81,82,83]. Moreover, chitosan’s chemical structure resembles glycosaminoglycans, which are the primary component of several human and animal tissues [41].

Chitosan-based scaffolds have been successfully tested in the regeneration of bones, tendons, cartilage, and periodontal tissue [41,80,81]. Due to the unique properties beneficial for wound-healing mentioned above (see Section 4.1.1. Chitosan in wound management) that include activation of cell growth, differentiation, and proliferation factors—enhancing the production of cytokines and cell migration—and stimulation of collagen production, chitosan seems a perfect material for such purposes. Chitosan biodegradability (e.g., by lysozymes) allows its reduction to oligomers after fulfilling its role in tissue regeneration [81].

However, quick biodegradation under acidic conditions in animal bodies where lysozymes are present is, at the same time, one of the most challenging disadvantages of chitosan scaffolds together with insufficient mechanical strength [84,85]. To overcome this problem, chitosan is often combined with other polymers, including collagen, alginate, gelatin, and hyaluronic acid or bioceramics such as hydroxyapatite and calcium phosphate, which provide the chitosan-based scaffolds with improved strength and biological properties [41,79,86,87].

#### 4.1.3. The Analgesic Properties of Chitin and Chitosan

Chitin and chitosan were shown to have a dose-dependent analgesic effect on inflammatory pain in mice when applied mixed with 0.5% acetic acid, reducing the abnormal behaviors due to pain, such as leg extension or abdominal torsion [88]. The effect of chitosan was greater compared to chitin due to its polycationic nature. The proposed mechanisms are that the analgesic effect of chitosan is based on its ability to absorb protons released in the inflammatory site, while chitin absorbs bradykinin, a substance related to pain [88,89].

Analgesic effect was also confirmed for carboxymethyl chitosan and O-carboxymethylated chitosan applied on scalded rats and albino rats with carrageenan-induced inflammations. The observed effect results from a reduced concentration of algogenic substances (bradykinin and 5-hydroxytryptophan) by using chitosan derivatives [90,91].

#### 4.1.4. Chitosan as a Drug Delivery Vehicle

Veterinary medicine needs reliable systems for the delivery of various chemotherapeutics, such as anesthetics, antibiotics, antiparasitic, painkillers, or growth promoters to mucosal epithelium for a local or systemic activity to avoid oral administration intended for intestinal absorption, which requires special formulations to avoid drug degradation in animal stomachs [34]. Since chitosan enhances the permeability of different skin layers, it makes an interesting material for transdermal drug delivery systems. Its potential for such purposes has already been shown in companion animals and ruminants [92,93,94,95].

Chitosan, independently or in combination with other chemicals, has been successfully tried as a delivery system for various macromolecules, including all kinds of drugs, peptides, and DNA [34,96,97]. It proved particularly useful in chemotherapy in animals for toxic drugs while delivering in other ways, for example, the heart-toxic anticancer doxorubicin or those with relatively short half-life and duration of action, like opioids [98,99,100]. It may also help deliver various immunomodulatory substances, including vaccines, anti-inflammatory agents, or adjuvants that would allow for animal disease control without using chemotherapeutics that are not acceptable for environmental concerns or are less effective due to increasing pathogen resistance [34,101,102].

#### 4.1.5. Other Applications of Chitosan in Veterinary Medicine

The immunomodulatory effect of chitosan and its derivatives, together with their antimicrobial properties and ability to remove toxins from animal bodies, make them an excellent material for biostimulant production—for example, in beekeeping—or as a dietary agent enhancing resistance against infections—for example, in fish [103,104].

### 4.2. Cellulose

Cellulose is the most abundant organic polymer and polysaccharide in nature. It is the main structural component of plants, some algae, and oomycetes and an ingredient of some bacteria secretions. In plant cell walls, cellulose is intertwined with hemicelluloses and lignin, while bacterial cellulose is relatively pure [105,106,107].

Chemically, cellulose is a linear polymeric chain composed of several hundred to thousands of β(1→4) linked D-glucose units. Each unit features six hydroxyl groups and two glycosidic bonds, which give the molecule hydrophilic properties and allow for specific chemical reactivity. Additionally, thanks to numerous hydroxyl groups, cellulose chains interact with each other through hydrogen bonds, forming microfibrils with high tensile strength. Fibrils consist of crystalline and amorphous regions. The properties of cellulose depend on its degree of polymerization and crystallinity [105,106,108].

**Figure 4:**
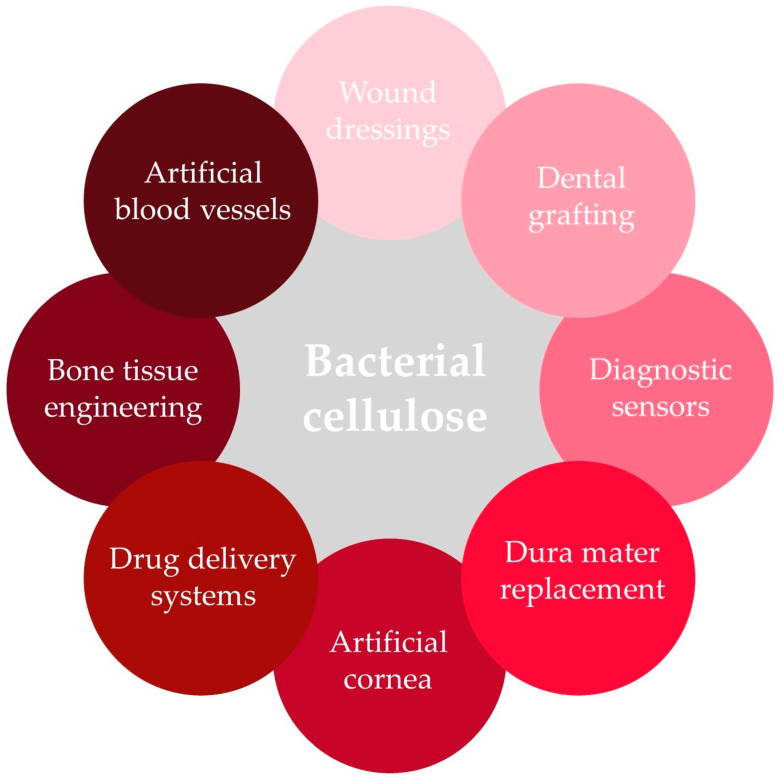
Bacterial cellulose in biomedical applications.

For medical applications, particularly bacterial cellulose (BC) is used, and several BC-based biomedical materials are already on the market [109]. Examples of bacterial cellulose applications in medicine are presented in Figure 4. Bacterial cellulose is produced mainly by *Gluconacetobacter xylinus* (*Acetobacter xylinum*) and is utilized due to its exceptional characteristics. It is an ultrafine material with high polymerization and crystallinity degrees; high surface area; water-holding capacity; and great moldability, flexibility, and tensile strength. Its high purity makes it a biocompatible, non-cytotoxic, and non-genotoxic material perfectly fitted for a broad range of purposes in human and veterinary medicine, including regenerative medicine, wound management, tissue engineering, implants, and drug carriers [107,108,109,110,111,112]. However, to improve its porosity and degradation rate and facilitate interactions with cells, bacterial cellulose is further chemically and/or physically modified using various in situ and ex situ methods [110]. Due to the fact that bacterial cellulose lacks antioxidant or antibacterial activities necessary for some biomedical materials and because pure cellulose hydrogels are difficult to store, handle, and maintain, BC is also often mixed with other natural or synthetic biomaterials, including chitosan, polylactic acid, graphene or graphene oxide to overcome those deficiencies [109].

#### 4.2.1. Cellulose in Wound Treatment

Pure crystalline cellulose in the form of a membrane proved a promising material for lesion healing in veterinary medicine. Easy to use, it has been successfully tried in the skin wound treatment in Wistar rats, decreasing healing time and pain, protecting the wound, maintaining its humidity, and enabling visualization and control of the lesion [113]. Additionally, a cellulose/graphene oxide nanocomposite tested on dorsum wounds in rats effectively promoted the healing process, increasing wound re-epithelization and neovascularization, as well as the formation of granulation tissue and collagen deposition while being cytocompatible [114].

The lack of antimicrobial properties limits cellulose’s usefulness in wound healing. Therefore, it is often combined or modified with various antimicrobial agents. The most common modifications are applied to bacterial cellulose because this is the purest cellulose type and thus the type most widely employed in wound management. One of the examples is bacterial cellulose hydrogel enriched with thymol, which showed excellent efficiency in third-degree burn healing in Wistar rats. Besides reducing the healing time of burn wounds, it also exhibited exceptional antimicrobial effectiveness against burn-specific pathogens [115]. Dressing materials based on BC are also often enriched with silver nanoparticles due to their antimicrobial potential. Promising results were obtained for a nanocomposite material made of polydopamine-coated BC with nano silver used for burn wounds treatment in rats [116] and for polyvinyl alcohol/bacterial cellulose hydrogel dressing loaded with silver nanoparticles applied for wound management in mice [117]. They were biocompatible; effectively promoted healing, reducing its time; and exhibited great antibacterial activity. Another dressing material for burn wound treatment was designed based on BC combined with montmorillonite and Cu/Na/Ca-modified montmorillonite. In tests on mice, they exhibited enhanced wound healing and tissue regeneration activity along with antibacterial effectiveness against typical burn wound pathogens so that they can be used as an artificial skin substitute in burn wound treatment [118].

To manufacture a novel modified Mohs paste, a treatment for malignant wounds, carboxymethyl cellulose was employed instead of zinc oxide starch powder to reduce the preparation efforts and metal-containing liquid waste produced when wounds are scrubbed. It effectively treated malignant wounds in dogs, reduced bleeding and malodor, and turned into gel on the wound surface, which facilitates its removal [119].

#### 4.2.2. Cellulose in Tissue Engineering

Tissue engineering uses biomaterials with the potential for tissue substitution and regeneration. Bacterial cellulose is particularly suitable for this purpose. Its nanostructure and morphology are similar to collagen, which makes it an attractive material for both hard and soft tissue scaffolds with various length scales, and it can promote tissue regeneration. Additionally, it can be combined with other polymeric and non-polymeric materials to gain proliferation, cell adhesion, and antimicrobial properties [108,112,120,121].

Pure BC scaffolds proved useful in bone regeneration in mice and rabbits [122]. Even better mechanical properties, biocompatibility, and osteoinductivity were obtained for scaffolds made of a combination of BC and gelatin, BC/gelatin/polylactic acid, or BC/gelatin/polylactic acid with hydroxyapatite coating, showing a future direction in developing artificial scaffolds for bone regeneration [122]. Besides bacterial cellulose, a carboxymethyl cellulose sheet loaded with calcium phosphate was also tested for bone regeneration in the dog lateral femoral condyle defect model. The new material was easily operable and effectively promoted new bone formation, proving its potential as a sheet-shaped bone graft substitute [123].

Bacterial-cellulose-based tissue-engineered blood vessels represent an innovative approach to overcoming reconstructive problems accompanying extended vascular diseases. Small-diameter BC tubes were used in several trials and applied to replace the carotid arteries in sheep and pigs or were attached to artificial defects of the carotid arteries of rats. The analyses revealed satisfactory bursting and suture retention strength of artificial vessels. An effective neoformation of a vascular wall structure along the BC scaffolds was observed, and the immigration of vascular smooth muscle cells into the synthetic matrix, without signs of inflammatory potential. The results confirm that the BC grafts provide stable vascular conduits promoting the neoformation of a vascular wall and are promising materials for future innovative programs for vascular surgery [121,124,125]. Moreover, their great potential for coronary and peripheral bypass grafting in pigs was also confirmed [126].

The high prevalence and incidence of both human and animal cardiovascular diseases, the leading cause of death worldwide, along with challenges regarding transplants, force the search for different therapeutic methods. Since traditional treatments cannot cure injured myocardium, using nanomaterials for heart regeneration is one of the most promising innovative approaches that have recently been tried. Nanostructural and morphological similarities to collagen make particularly a bacterial cellulose hydrogel a promising material for this purpose. Cellulose scaffolds (made of BC alone, cellulose acetate, or a combination of polyurethane and ethyl cellulose) proved to provide a three-dimensional structure to effectively support cell immobilization, facilitating their growth and proliferation, which makes them safe and innovative materials for cardiovascular repair in both human and animal medicine [109,127,128,129].

A bilayer nanofibrous potato starch/bacterial cellulose scaffolds with muscle cells cultured on them were tried as biomaterials in hollow organ reconstruction. They proved effective in repairing urethral defects in dogs. Due to the adjustable pore size and porosity, the composite scaffolds have the potential to be used in the reconstruction of various hollow organ tissues, including the bladder, esophagus, intestine, ureter, and vascular tissues [130].

Bacterial cellulose also seems to be a promising material for tissue-engineered cornea, which, due to the limited number of corneal donors, is needed for transplantations necessary to cure severe corneal diseases. BC scaffolds were successfully tested for corneal stroma replacement in rabbits, showing good biocompatibility and stability [131]. Moreover, a novel engineered corneal stroma made of bacterial cellulose and polyvinyl alcohol hydrogel tested on rabbits exhibited even better properties than a hydrogel made of BC only, showing its potential to substitute corneal stroma in both human and veterinary medicine [132].

#### 4.2.3. Cellulose-Based Drug Vehicles

To effectively cure various diseases in humans and animals, a controlled and sustained release of an accurate dose of drugs is necessary. Recently, a search for new effective delivery vehicles acquired from sustainable bioresources has been visible due to their higher biocompatibility, nontoxicity, and reduced side effects. One of the best biomaterials for this purpose is nanocellulose. Biocompatible, inert, and with excellent mechanical properties, it has a high surface-area-to-volume ratio and tunable chemical structure that allows various surface modifications to enhance binding with particular drugs and let their controlled and sustained release to the target. Many types of nanocellulose forms (aerogels, hydrogels, films, membranes, and nanoparticles) enable their use for drug delivery externally or internally [133,134]. The types of drug administration using cellulose vehicles include:

- Oral administration—This is the most common route due to the ease and cost-effectiveness combined with high patient compliance. However, its effectiveness is affected by physiological barriers and drug stability in the gastrointestinal tract; therefore, different new pharmaceutical technologies are developed to overcome the obstacles. One of the examples is the dual site-targeted and release dome matrix designed to have both gastric and intestinal targeting capacities. The caffeine-, melatonin-, and hydroxypropylmethylcellulose-based modules completely released melatonin in the stomach due to the favorable pH and effectively delivered caffeine to the colon. This reference oral carrier will be useful in veterinary medicine and other applications where a complex formulation and diverse in vivo performance are necessary [96];
- Local application—when the active chemical is delivered directly at or near the target site to avoid intoxication or damage to the surrounding tissue. One of the examples is ethyl cellulose–ethanol ablation, which was tried as an intratumoral injection in cats with sublingual squamous cell carcinoma to retain tumoricidal doses of ethanol within the tumor without damaging other tissues. Although tumor volume was reduced in some cats, concurrent lingual dysfunction occurred, excluding this kind of treatment from veterinary practice. However, further optimization of the applied treatment may make it an interesting minimally invasive option for curing oral, neck, and head cancers in cats and other animals [135];
- Transdermal drug delivery—This involves drug delivery through the skin, and in veterinary medicine, it is a useful alternative to more traditional oral drug administration since it is non-invasive; avoids the gastric route, reducing potential gastric irritation and drug degradation; and has a reduced first-pass metabolism in the liver [134,136]. Cellulose and its derivatives can serve as a base for hydrogels with dispersed active ingredients, which perform better than creams and ointments due to their better adhesion, cooling effect, ease of removal, excellent drug-loading efficiency, and improved drug release. For example, alaptide, used commercially in the form of cream in wound management in veterinary practice due to its regenerative properties and enhancement of epithelization processes, was tried as a formulation in hydrogels made of methylcellulose, hydroxyethylcellulose, and hydroxypropylcellulose. The results showed that alaptide incorporated into 3% hydroxyethylcellulose hydrogel exhibited the best properties and was appropriate for veterinary practice [137]. Another example is cationic hydroxyethyl cellulose surface-modified MoS_2_ nanoparticles with excellent photothermal conversion abilities as a transdermal drug delivery system. It was successfully tried for the model drug atenolol in rabbits, showing great skin penetration without irritation. Although the high toxicity of MoS_2_ limits its biological application, the study showed the potential of such systems for delivering small molecular drugs in animals [138].

The commercialization of new applications of cellulose as a drug carrier requires further in vitro and in vivo studies along with clinical trials, but the functionalities and applicability offered by this polymer are promising, showing a new path in drug administration in future veterinary medicine [134].

### 4.3. Poly(ε-caprolactone) (PCL)

Poly(ε-caprolactone) (PCL) is a semi-crystalline biodegradable aliphatic ester with a glass transition temperature of about −60 °C and a low melting point of around 60 °C. The polymer can be obtained by ring-opening polymerization of ε-caprolactone in the presence of various catalysts. Its relatively slow hydrolytic degradation makes it a perfect polymer for long-term medical applications lasting more than a year. PCL is often used in combination with other polymers, including polylactic acid and polylactic acid-co-glycolic acid, to enhance biodegradation, improve adhesion and stress crack resistance, or to manipulate the rate of drug release from PCL microcapsules [139,140].

#### 4.3.1. Tissue Engineering Using Poly(ε-caprolactone)

Similarly to the polymers mentioned above, PCL can serve as a base for scaffolds useful in tissue regeneration. One of the promising composites for this purpose is polycaprolactone-tricalcium phosphate (PCL-TCP). It was successfully tried as an implant in dogs with distal radial osteosarcoma in a limb-sparing surgery. As a result, a significant improvement in limb function was observed without any complications due to the scaffold itself or the surgery, showing the potential of the composite in surgeries in animal patients with bone tumors [141]. Although intended for human medicine, polypropylene mesh functionalized with PCL nanofibers was also tested on animal models as a scaffold for incisional hernia repair or prevention since their structure mimics natural extracellular matrix. Experiments on rabbit and minipig models gave promising results, showing accelerating collagen maturation and leaving a scar that is more elastic and flexible than a standard mesh, thus opening a new perspective for hernia treatment and prevention [142,143]. A bilayer oxidized regenerated cellulose/poly ε-caprolactone composite was successfully applied for a dural closure after a neurosurgical procedure in the rabbit model due to its biocompatibility and regenerative properties, resulting in great osteogenesis [144]. PCL also proved to be a suitable biomaterial for producing screws used in the experimental surgical management of a partial anterior cruciate ligament rupture in animals [145].

The PCL-based scaffolds can be loaded with appropriate cells to enhance their regenerative performance. Seeded with mesenchymal stem cells, the poly(ε-caprolactone) scaffolds proved effective as an alternative meniscal substitution in tests on rabbits, showing improved fibrocartilaginous tissue regeneration and mechanical strength and lower cartilage degeneration compared to cell-free PCL scaffolds [146], while PCL-TCP scaffolds helped in healing critical bone size defects in a dog’s mandible, thus showing their potential for bone regeneration [147].

#### 4.3.2. Poly(ε-caprolactone) as a Drug Delivery Vehicle

Poly(ε-caprolactone)’s slow degradation, biocompatibility, high porosity, and nontoxicity make it a good material for drug delivery in various forms, especially for drugs with local action or high general toxicity, which are difficult to deliver or have to be released into specific places in the animal body. One of the examples is novel chloramphenicol loaded with poly(ε-caprolactone)-pluronic composite nanoparticles, which bypasses the problem of toxicity, poor penetrability, and fast degradation of free chloramphenicol and enables its application for the treatment of MRSA-infected burn wounds as shown on an animal model. The use of PCL-based nanoparticles enhanced chloramphenicol’s bioavailability and therapeutic activity while reducing its toxicity, showing the great potential of such a novel approach for delivering effective but problematic antimicrobial drugs in human and veterinary medicine [148]. Another example is a fast-degrading PLC-based intraocular implant with enhanced porosity loaded with dexamethasone. Non-toxic to retinal cells, it did not alter the structure and function of the retina, demonstrating its usefulness as a drug vehicle in retinal diseases [149]. Poly(ε-caprolactone) also proved effective for producing intravaginal inserts for the delivery of progesterone to cattle [150].

### 4.4. Polylactic Acid (PLA)

Polylactic acid, also known as poly(lactic acid), poly lactic acid, or polylactide (PLA), is a linear aliphatic thermoplastic polyester (Figure 2) obtained by direct condensation of lactic acid or polymerization by ring-opening of acid esters. It forms a long linear chain composed of repeated monomer units of lactic acid. The monomers are produced from fermented plant starch sourced from sugarcane, corn, or sugar beet pulp and exist in two enantiomeric forms of L and D isomers, where L-isomer is a dominant form produced in fermentation processes [151,152,153].

PLA is a biodegradable, biocompatible, renewable, nontoxic, eco-friendly polymer and is one of the most common industrially used biopolymers with a wide range of uses, including packaging materials, filament material for 3D printing, and the production of nonwoven fabrics. Since PLA also has good biological safety, mechanical resistance, and processability profiles with antibacterial, flame-retardant, and oil- and water-resistant properties, it also has the potential to be applied as an alternative to high-cost, non-biodegradable biocompatible materials for biomedical purposes. So far, it has been used as a degradable suture, bone fixation device, orthopedic device, material for surgical implants, drug delivery systems, and scaffolds for tissue engineering applications. However, for some purposes, the low hardness of PLA is insufficient, which limits its usability; therefore, extensive research has been conducted to develop various co-polymers with improved performance to overcome this disadvantage, combining PLA with polycaprolactone and hydroxyapatite [140,151,152,154,155,156].

#### 4.4.1. PLA for Veterinary Orthopedic Surgeries

Traditional metallic implants used in veterinary medicine are problematic, causing bone resorption, infections, and pain at the application site, and their removal poses a risk and is expensive. Therefore, there is a need to develop new types of safer and cheaper implants, and PLA seems to be a promising material for such applications. Additionally, the development of three-dimensional (3D) printing technology allows the preparation of personalized implants with a similar anatomical structure to real bones made of biocompatible and biodegradable polymers, which will decrease the costs of orthopedic surgeries and enable to adapt implants to individual patients [157,158,159,160,161].

PLA implants have already been tested in clinical trials with dogs. They proved effective in treating tibial tuberosity advancement, safe for veterinary patients, and provided good functional results. They showed faster ossification, facilitating more efficient bone healing of osteotomy gaps, and had similar clinical recovery time compared to metallic implants, presenting an acceptable rate of complications [157,158]. Additionally, PLA or PLA-based bone fixation plates, nails, and screws proved a better alternative to their metallic equivalents, providing enough support to the fracture during healing and then slowly degrading when no longer needed [162,163,164,165].

Another complex problem in veterinary orthopedics is tissue regeneration in critical-size bone defects, and there is an urgent need to find reliable osteoconductive biomaterials for this application. It turned out that PLA, individually or in mixtures with other biodegradable polymers, is suitable for a 3D printing technique that enables the production of scaffolds that may help treat large bone defects [166]. Polylactic acid/polycaprolactone/hydroxyapatite (PLA/PCL/HA) scaffolds loaded with bone marrow stem cells or differentiated bone cells proved effective in bone regeneration in Wistar rats with critical bone defects. The results of immunohistochemical, histopathological, histomorphometric, and biomechanical analyses demonstrated significantly enhanced bone healing in both scaffold and autograft groups compared to the untreated group, and the effects of using cell-loaded scaffolds were similar to the autograft [167,168,169]. Moreover, PLA or PLA/PCL/HA scaffolds can also be loaded with osteoinductive drugs such as purmorphamine or nandrolone to increase healing quality and shorten bone regeneration time effectively [170,171]. Additionally, PLA scaffolds combined with platelet-rich plasma proved effective in completely healing the hoof wall defect caused by keratoma in horses [172].

#### 4.4.2. PLA in Drug Delivery Systems

Veterinary medicine requires unique, long-lasting drug formulations to overcome problems with drug bioavailability and other adverse effects, and polymers, like PLA, seem suitable carrier alternatives due to their prolonged biocompatibility and biodegradability [154]. Several clinical trials have already been conducted to evaluate the usefulness of PLA as a drug delivery vehicle.

The therapeutic effects of PLA microspheres loaded with lactones from *Venenum Bufonis* on mycoplasma pneumoniae were tested on swine, where the microspheres combined with tylosin and florfenicol were injected intramuscularly. The results demonstrated a significant drug slow-release effect by the microspheres, efficiently reducing drug toxicity and enhancing its activity time [173].

Metformin helps treat diabetes, but when applied individually, it has a large variability in gastrointestinal absorption and bioavailability dependent on the patient species. Therefore, metformin-loaded PLA microparticles were tested for diabetes treatment in rabbits to evaluate if PLA can improve the metformin pharmacokinetics profile. The results show that the obtained microparticles have optimal physicochemical characteristics and allow for the prolonged/sustained release expected for metformin, which makes them potentially suitable for diabetes treatment in animals [174]. Curcumin-nisin-based PLA nanoparticles were also successfully tested for cardioprotection in guinea pigs with induced myocardial infarction [175].

Electrospinning and solution blow spinning technologies enable the production of novel biomaterials loaded with active agents for the medical treatment of humans and animals. Some examples include nanofibrous PLA mats loaded with progesterone, which proved a promising alternative for the controlled delivery of progesterone to control the estrus cycle in livestock animals [176], or PLA/nano-hydroxyapatite/doxycycline fibers that proved effective against common periodontal pathogens *Aggregatibacter actinomycetemcomitans* and *Porphyromonas gingivalis*, showing higher antimicrobial effects compared to antibiotics traditionally used in similar cases and being an alternative for non-surgical local treatment in periodontitis [177].

Biocompatible PLA nanoparticles are promising carriers and adjuvants for newly developed mucosal vaccination in human and veterinary medicine. As shown in tests on zebrafish, they are efficient and versatile in carrying immune-stimulating molecules and antigens and targeting antigen-presenting cells, thus showing great potential in applying mucosal vaccines for humans and aquacultures [178].

#### 4.4.3. PLA for Wound Management

PLA characteristics make it a potentially useful material for wound dressings. Dressing mats prepared from a blend of hydrophobic PLA fibers with hydrophilic cellulose acetate or poly(ethylene oxide) and loaded with antibacterial and anti-inflammatory sulfonamide analog were tested on mice. Compatibility of the drug with nanofibers was shown, as well as a reactivity between PLA and cellulose acetate that limits its leaching from the polymer matrix. The mats proved effective in enhanced wound healing resulting from enhanced anti-inflammation, neo-angiogenesis, epithelization, fibroplasia, and collagen deposition at the laceration area [179].

Due to its hemostatic properties, PLA can also serve as an effective and nontoxic hemostatic agent for areas difficult to access, such as bleeding occurring during minimally invasive surgeries. Nonwoven PLA fabric manufactured by Toray Industries, Inc. was tested on rats for its efficacy in shortening bleeding time in liver hemostasis, thus confirming its potential for such purposes in human and veterinary medicine [180].

### 4.5. Poly(lactic-co-glycolic Acid) (PLGA)

Poly(lactic-co-glycolic acid) is an aliphatic amorphous polymer (Figure 2) synthesized as either random or block copolymer by means of ring-opening co-polymerization or direct polycondensation reaction of two monomers: lactic acid (LA) and glycolic acid (GA). The molecular weight of the polymer and LA/GA ratio determine its physicochemical properties, enabling the control of the degradation kinetics—the higher the GA content, the faster the degradation [181]. The controllable biodegradability, biocompatibility, and superior mechanical properties of PLGA make it a suitable biomaterial for medical purposes, particularly for drug delivery systems and tissue engineering.

#### 4.5.1. PLGA as a Drug and Vaccine Carrier

Modern medicine searches for delivery systems that enhance the therapeutic effects of applied drugs while reducing their potentially unwanted or dangerous side effects. Biodegradable and non-toxic poly(lactic-co-glycolic acid) broken down in the body into GA and LA and eventually metabolized into carbon dioxide and water is a perfect biomaterial for this purpose. Changing the LA to GA ratio and molecular weight of the polymer makes it possible to tailor its properties, including the rate and speed of degradation and drug release mechanisms, to the needs of individual applications. Additionally, specific functionalization of its surface can restrict or limit drug entry into healthy cells, increasing the precision and safety of the treatment [181,182,183].

In veterinary medicine, PLGA microparticles have been successfully tried, among other things, as a vehicle for short-acting opioid drugs such as buprenorphine to prolong its analgesic effect and limit the number of injections, showing great potential for future commercialization and industrial production [184]. PLGA nanoparticles were also tested in combination with lutein as an ophthalmic nanodelivery system [185], making an effective ivermectin delivery matrix useful in the prevention of heartworm disease in dogs [186].

Poly(lactic-co-glycolic acid) nanoparticles, although used alone, are relatively ineffective in inducing a strong immune response, in combination with other compounds, they make a promising vaccine delivery system. While applied with effective immunomodulators such as Chinese yam polysaccharide or influenza virus conserved peptides, they can produce a strong humoral and cellular immune response, laying the foundation for novel adjuvant design [187,188,189]. Surface modification of PLGA nanoparticles with cationic polymers (e.g., polyethylenimine, chitosan, ε-Poly-L-lysine) enhances their adjuvanticity and antigen loading efficiency, enhancing stability and enabling the induction of high immune response by antigen-presenting cells, showing their great potential as antigen delivery systems/vaccine carriers [189,190].

#### 4.5.2. Tissue Engineering Using PLGA

PLGA is a versatile biomaterial that can be formed not only into micro- or nanoparticles but also membranes, films, and scaffolds of various shapes. Its mechanical properties and biodegradation time can be manipulated by controlling the lactic-to-glycolic acid ratio, making it a perfect material for tissue engineering applications in both human and veterinary medicine. Numerous studies have proven that PLA is suitable for three-dimensional (3D) printing with fused deposition modeling technology to manufacture scaffolds used in critical-sized bone defects [191], for soft and hard tissue healing [192,193], and in the dental field [194,195]. Additionally, PLGA-based scaffolds loaded with bone marrow mesenchymal cells proved effective in the regeneration of various types of tissues, including regeneration and functional recovery of the sciatic nerve in dogs, opening a new field in such needed and rapidly developing tissue regeneration area [196,197].

### 4.6. Polymers Synthesized from Bacteria

Polyesters derived from bacteria are naturally occurring biodegradable polymers. There are two major polyesters in this category: polyhydroxyalkanoates (PHA) [198] and polyhydroxybutyrate (PHB) [199]. Polyhydroxyalkanoates (Figure 2) are natural polyesters of 3-, 4-, 5-, and 6-hydroxyalkanoic acids with molecular weight ranging from several thousand to several million Daltons. Various microorganisms produce them as lipid inclusions and store them within their cells as a carbon and energy source. PHAs differ in chemical composition and the resulting properties due to the variations in building monomers. They are insoluble in water and resistant to hydrolytic attack; they are also biodegradable, where degradation depends on their chemical composition, environmental conditions, and the type of degrading microorganism [200,201].

Bacterial polyesters are considered green plastic with a positive environmental and social impact compared to conventional petrochemical-based plastic regarding their production and recycling. Nontoxicity, biocompatibility, and biodegradability along with controllable biodegradability and adjustable thermal and mechanical properties make them—in particular, poly-3-hydroxybutyrate (PHB), poly-4-hydroxybutyrate (P4HB), poly-3-hydroxyoctanoate (PHO), copolymers of 3-hydroxybutyrate and 3-hydroxyvalerate (PHBV), and copolymers of 3-hydroxybutyrate and 3-hydroxyhexanoate (PHBHHx)—excellent renewable and sustainable materials for multiple applications in the biomedical sector, including wound dressing, tissue engineering, artificial organ construction, drug delivery, and bio-implants. However, despite their outstanding characteristics, they still cannot replace traditional plastic because of the high production costs, which are 5–10 times higher than petroleum-based plastics [200,202,203].

#### 4.6.1. PHA and PHB for Tissue Engineering

Biopolymer composites enable the establishment of an ideal environment for regenerating chondral, osteochondral, and osseous defects in articular cartilage and bones in cases where their natural ability to regenerate is impaired. They provide excellent stabilization at the beginning of the treatment, and with time, their biodegradability allows for a gradual loss of physical resistance and, eventually, the resorption of the implant, thus facilitating the regeneration of the defect. With structural, chemical, and physical properties similar to bone or cartilage, particularly polyhydroxybutyrate-based composites have emerged as an ideal material for producing resorbable orthopedic implants [204,205,206]. PHB, especially when blended with 3-hydroxyvalerate to make P(HB-HV), has seen more attention in orthopedic applications because of its piezoelectric properties that aid in the healing of bones [207].

One of the examined composites was polyhydroxybutyrate/chitosan. Implants made of this material tested on the sheep animal model with knee cartilage defects proved effective in facilitating the healing process, showing their potential for treating chondral and osteochondral defects of traumatic character [206]. Porous polyhydroxybutyrate/chitosan scaffolds seeded with mesenchymal stem cells tried for reparation of knee cartilage defects in sheep successfully supported the formation of cartilage-like tissue and wound healing [208]. In turn, polyhydroxybutyrate/hydroxyapatite composites used as implants in animals with bone defects proved to be biocompatible, biodegradable, osteoconductive, and effective in integrating into bone; however, in some cases, they triggered a chronic local inflammatory response [204,205,209]. PLGA/PHB implants reinforced or not with growth factor IGF1 intended for osseous tissue regeneration and tested on animal models showed high biocompatibility and conduced tissue regeneration without allergic or other adverse reactions [193,210]. Although further studies are still needed to develop effective and safe PHA composite-based implants and scaffolds, the results obtained for all the experimental implant prototypes mentioned above promise a new sustainable era in human and animal tissue engineering.

#### 4.6.2. PHA/PHB-Based Drug Carriers

Veterinary medicine is still facing challenges related to safe, effective, and low-cost methods and routes of drug administration due to differences between various animal species; hence, there is a search for new drug carriers among biodegradable polymers.

Reproductive management of cattle requires estrus synchronization, where progesterone is a commonly used steroid hormone. One newly tested strategy is its administration through ear implants made of progesterone encapsulated in biopolymer nanoparticles, with poly-hydroxy-butyrate and valerate (PHB-V) as main components, also in combination with PCL. In vitro tests showed that the size of PHB-V nanoparticles and their associations with PCL affect the quantity and the kinetics of drug release [211], which will help develop appropriate drug-loaded implants. In vivo tests using intravaginal progesterone-loaded PHB/PCL implants in cows confirmed the usefulness of these polymers as drug carriers with adjustable drug release rates and biodegradation time [212]. Additionally, other PHB-based composites such as P(HB-HV) have been shown to be ideal for drug delivery since degradation is by surface erosion [18].

Bovine tuberculosis is one of a major health problem in cattle and development of an effective vaccine to control this disease is of great importance. PHB nanoparticles which displayed mycobacterial antigens on the surface of the bio-beads were successfully tried for this purpose, showing the potential of this approach to produce cost-effective vaccines to combat tuberculosis or other diseases in animals [213].

Bacterial polyesters can also be used as dietary supplements. Aquacultures require effective disease control agents to maintain farmed aquatic animals in good health and condition. PHB was found to serve as a suitable biocontrol compound since it can be degraded into short-chain fatty acids (SCFAs), the antimicrobial properties of which are well-recognized [214]. When tried as a bioactive agent in bioflocs—used as a dietary supplement in gibel carp and European sea bass breeding—PHB proved to effectively enhance immunity and disease resistance of farmed animals and significantly increased the number of beneficial bacteria such as *Bacillus* sp., which are beneficial for their health. PHB supplementation also increased the fish weight [215,216]. Pacific white shrimp fed with PHB and butyrate-supplemented diet showed improved the digestive capacity, growth and immune function [217,218]. Similar effects were obtained with PHB-fed Nile tilapias and Siberian sturgeons [219,220].

## 5. Conclusions

**Figure 5:**
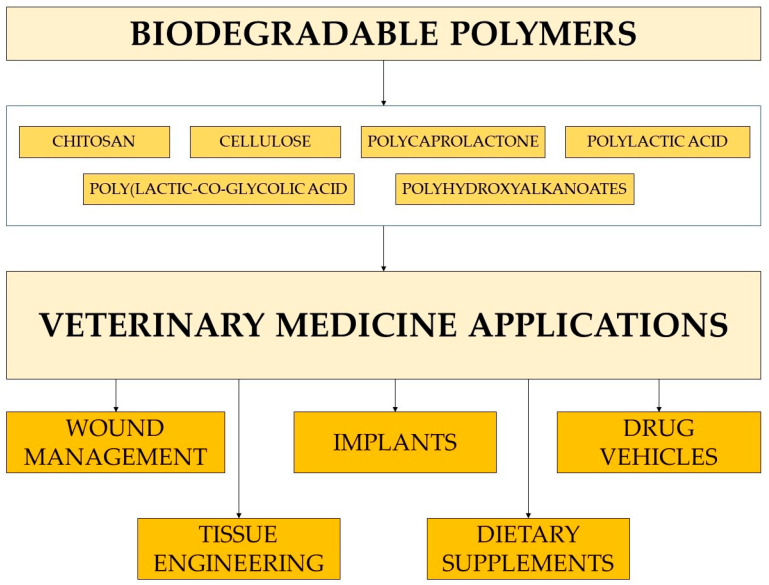
Main areas of biodegradable polymers applications in veterinary medicine.

Biodegradable polymers are the preferred biomaterial for the development of therapeutic devices, including implants, tissue engineering, and controllable drug carriers (Figure 5). The polymers must be designed to work specifically towards the desired outcome, whether it be facilitating new tissue growth, possessing antimicrobial resistance, having mechanical properties that address short-term functionality, having negligible toxicity, and delivering drugs to their targeted location upon degradation. For veterinary medicine, it is also crucial that the biomaterials be highly effective but low in cost.

This review summarizes some natural and synthetic biodegradable polymers that exist commercially that are most applicable to veterinary medicine. Of the enzymatically degradable natural polymers, the aminoglycans (e.g., chitin and chitosan) and modified celluloses have tremendous potential. Of the hydrolytically degradable synthetic thermoplastic polymers, polyesters have shown immense diversity and versatility, including polylactic acid, poly(lactic-co-glycolic acid), and polyhydroxyalkanoates.

Chitosan has shown many advantages, especially in wound management through activating cell growth factors while exhibiting no adverse effects on animal tissue. Other advantages include analgesic effects to decrease inflammatory response and allows for transdermal drug delivery. One major disadvantage of chitosan is that biodegradation occurs rapidly under acidic conditions, so other natural polymers need to be grafted with chitosan to improve strength and degradation resistance.

Cellulose, particularly bacterial cellulose, is well-suited for a variety of therapeutic applications. However, it requires chemical and physical modification to be useful. Therefore, it is typically grafted with other biomaterials and enriched in antimicrobial compounds and used extensively in wound treatment and tissue scaffolding. Furthermore, nanocellulose in its many forms aids in drug delivery both externally and internally.

Poly(ε-caprolactone), because of its slow degradation, makes an excellent long-term solution for tissue scaffolding in hernia repair and in drug delivery when release needs a specific location. Polylactic acid, because of its low hardness, is typically co-polymerized with poly(ε-caprolactone) and hydroxyapatite. Of its many advantages, 3D printing of personalized implants increases biocompatibility, which decreases orthopedic surgery costs significantly and can be incorporated with osteoinductive drugs. Polylactic acid also allows for long-lasting drug formulations in drug delivery systems, unique to veterinary medicine. Poly(lactic-co-glycolic acid) has the advantage of controllable degradation kinetics with altering the lactic acid/glycolic acid ratio. This is particularly beneficial in veterinary medicine to prolong analgesic effects so as to limit injection frequency.

Of the polymers synthesized by bacteria, polyhydroxybutyrate composites have become ideal in orthopedic implants, particularly due to their piezoelectric properties. However, more research is needed to further develop some of these composites without causing inflammation. One promising area for these composites is drug delivery and using them as low-cost drug carriers.

The usefulness of biodegradable polymers in veterinary medicine for the benefit of both animal patients and the environment is indisputable. Although many of the described studies have been performed only at the experimental level and further research is still needed for many polymers to enter clinical veterinary practice, the future seems promising. It is worth noting that, except for therapeutics dedicated specifically to veterinary medicine, many applications intended for human treatment are also tested on animals. Positive results and progress in these human-focused studies enable widespread use of newly developed polymer-based materials in veterinary medicine as well, but only if the biomaterial application systems are made sufficiently effective and economical. This new era of specifically designed biodegradable polymers, emerging for the benefit of all on earth, allows for even greater tailoring of polymers to match the therapeutic application.

## Figures and Tables

**Table 1:** Examples of polymers which are the most often used in medicine [6,7,8].

Polymer Type	Application
polyolefins	sliding surfaces of artificial joints
poly(tetrafluoroethylene)	a vascular graft
poly(vinyl chloride)	extracorporeal tubing or blood storage bags
silicone	useful in ophthalmologic applications, fibrous capsule formation at breast implants
methacrylates	applied in dentistry and orthopedics
polyethers	for orthopedic applications and dialysis membranes
polyesters	available in different shapes, from solid materials for orthopedic applications via meshes to drug-eluting coatings on vascular stents
polyamides	for suture materials
polyurethanes	for urinary catheters and ureteral stents

## Data Availability

No new data were created.
